# Complete Chloroplast Genome Sequence of *Aquilaria sinensis* (Lour.) Gilg and Evolution Analysis within the Malvales Order

**DOI:** 10.3389/fpls.2016.00280

**Published:** 2016-03-08

**Authors:** Ying Wang, Di-Feng Zhan, Xian Jia, Wen-Li Mei, Hao-Fu Dai, Xiong-Ting Chen, Shi-Qing Peng

**Affiliations:** ^1^Key Laboratory of Biology and Genetic Resources of Tropical Crops, Ministry of Agriculture, Institute of Tropical Bioscience and Biotechnology, Chinese Academy of Tropical Agricultural SciencesHaikou, China; ^2^College of Agronomy, Hainan UniversityHaikou, China; ^3^State Key Laboratory of Cellular Stress Biology, School of Life Sciences, Xiamen UniversityXiamen, China

**Keywords:** *Aquilaria sinensis* (lour.) gilg, chloroplast genome, simple-sequence repeat, relative synonymous codon usage, phylogenetic analysis

## Abstract

*Aquilaria sinensis* (Lour.) Gilg is an important medicinal woody plant producing agarwood, which is widely used in traditional Chinese medicine. High-throughput sequencing of chloroplast (cp) genomes enhanced the understanding about evolutionary relationships within plant families. In this study, we determined the complete cp genome sequences for *A. sinensis*. The size of the *A. sinensis* cp genome was 159,565 bp. This genome included a large single-copy region of 87,482 bp, a small single-copy region of 19,857 bp, and a pair of inverted repeats (IRa and IRb) of 26,113 bp each. The GC content of the genome was 37.11%. The *A. sinensis* cp genome encoded 113 functional genes, including 82 protein-coding genes, 27 tRNA genes, and 4 rRNA genes. Seven genes were duplicated in the protein-coding genes, whereas 11 genes were duplicated in the RNA genes. A total of 45 polymorphic simple-sequence repeat loci and 60 pairs of large repeats were identified. Most simple-sequence repeats were located in the noncoding sections of the large single-copy/small single-copy region and exhibited high A/T content. Moreover, 33 pairs of large repeat sequences were located in the protein-coding genes, whereas 27 pairs were located in the intergenic regions. *Aquilaria sinensis* cp genome bias ended with A/T on the basis of codon usage. The distribution of codon usage *in A. sinensis* cp genome was most similar to that in the *Gonystylus bancanus* cp genome. Comparative results of 82 protein-coding genes from 29 species of cp genomes demonstrated that *A. sinensis* was a sister species to *G. bancanus* within the Malvales order. *Aquilaria sinensis* cp genome presented the highest sequence similarity of >90% with the *G. bancanus* cp genome by using CGView Comparison Tool. This finding strongly supports the placement of *A. sinensis* as a sister to *G. bancanus* within the Malvales order. The complete *A. sinensis* cp genome information will be highly beneficial for further studies on this traditional medicinal plant. Moreover, the results will enhance our understanding about the evolution of cp genomes of the Malvales order, particularly with regard to the role of *A. sinensis* in plant systematics and evolution.

## Introduction

Plant chloroplasts (cps) are key organelles for photosynthesis and carbon fixation (Neuhaus and Emes, 2000). The cp genome sequence contains useful information in plant systematics because of its maternal inheritance in most angiosperms (Corriveau and Coleman, 1988; Zhang et al., 2003). Substitution rates in plant cp genomes are much lower than those in nuclear genomes (Wolfe et al., 1987). Plant cp genomes are valuable sources of genetic markers for phylogenetic analyses because of their very low level of recombination (Provan et al., 2001; Ravi et al., 2008). The cp DNA sequence was initially discovered during physical mapping of the *Zea mays* cp, which was produced by digestion with multiple restriction enzymes (Bedbrook and Bogorad, 1976). Subsequently, the first complete nucleotide sequence of *Nicotiana tabacum* was determined by the clone sequencing of plasmid and cosmid libraries (Kumano, 1986). Over 600 plant cp genomes have been sequenced and deposited in the NCBI Organelle Genome Resources. The cp genome evolution in land plants may be elucidated using these database resources. The cp in angiosperms exhibits a conserved quadripartite structure ranging from 115 to 165 kb in length and consists of one large single-copy (LSC) region, one small single-copy (SSC) region, and two copies of inverted repeat (IR; Palmer, 1991; Raubeson and Jansen, 2005). The *Arabidopsis thaliana* cp genome contains a circular DNA composed of 154,478 bp with 87 potential protein-coding genes (Sato et al., 1999). The cp DNA from maize (*Z. mays*) consists of 140,387 bp with a total of 104 genes (Bedbrook and Bogorad, 1976). The complete cp DNA of *Cedrus deodara* is circular molecule of 119,298 bp with 114 genes (Ching et al., 2010). However, some parasitic plants, such as *Conopholis americana*, which demonstrate unique life cycles, are exceptions because the sizes of their cp genomes are beyond 115–165 kb, with the smallest plastome of 45 kb in land plants (Wicke et al., 2013). The development of DNA sequencing technology has resulted in the extensive use of cp genomes for molecular marker and molecular phylogenetic studies (Tangphatsornruang et al., 2009; Takano and Okada, 2011; Awasthi et al., 2012; Jheng et al., 2012; Chen and Melis, 2013; Turner et al., 2013; Gaudeul et al., 2014).

Agarwood is widely used as a sedative, digestive, and antiemetic traditional drug. Agarwood sculpturing is valuable for interior decoration and is also popularly used as incense and perfume in Asia. The stems, branches, or roots of *Aquilaria* and *Gyrinops* trees are wounded and infected by fungi to produce agarwood (the wounds can be caused by wind, lightning strikes, gnawing of ants or insects, or microorganism invasion). *Aquilaria sinensis* is the only certified source for producing agarwood listed in China Pharmacopoeia (China pharmacopoeia Committee, 2010). All *A. sinensis* species are endangered because of the high demand for agarwood products; hence, these species are regulated under the Convention on International Trade in Endangered Species of Wild Fauna and Flora. However, the genomic resources for *A. sinensis* are limited, and little is known about the composition and organization of its cp genomes and their evolution. In this study, we report the complete cp genome sequence of *A. sinensis* (GenBank accession number: KT148967) in accordance with the Illumina Hiseq2500 standard protocol. Overall, the results provide basic genetic information on *A. sinensis* cp and the role of *A. sinensis* in plant systematics and evolution.

## Materials and methods

### DNA extraction and sequencing

*Aquilaria sinensis* fresh leaves were collected from a 2-year-old tree at the Experimental Farm of the Chinese Academy of Tropical Agriculture Sciences, Hainan, PR China. The leaves were cleansed, frozen in liquid nitrogen, and ground using a tissue lyser. DNA was extracted using a Plant Genomic DNA Kit (Foregene Biotech, China). DNA was used to generate 500 bp (insert size) paired-end library in accordance with the Illumina Hiseq2500 standard protocol. Approximately 3.1 Gb of raw data were generated with pair-end 125 bp read length.

### *De novo* CP genome assembly

The obtained nucleotide sequencing reads were qualitatively assessed and assembled to contigs by using SOAPdenovo2 (Luo et al., 2012) with kmer length of 83. The assembled contigs included a mixture of sequences from organellar and nuclear genomes. The average coverage of cp genomes is usually much higher than that of nuclear genomes because many cps are found in a single cell (Steele et al., 2012; Straub et al., 2012). Thus, a complete *de novo* assembly of the cp genomes was performed using the assembly quality-filtered reads that exhibit high coverage for the cp genomes. We sorted the assembled contigs by contig-read depth analysis of assemblies by using the high correlation between sequencing depth and number of copies in the genome. The quality-filtered reads were remapped to the assembled contigs to calculate the sequencing depth with BWA (Li and Durbin, 2009). Thus, the cp contigs with high coverage (more than 500 ×) were isolated from the nuclear contigs by using the difference of read depths between contigs (Figure S1). All published cp genome sequences of dicotyledons were used as references to map the contigs with BLAST (Table S1) and thus confirm the cp genome contigs. Finally, all isolated cp contigs were combined, and reads were recaptured to isolate more cp DNA reads. Contigs were reassembled and extended to obtain a complete cp genome sequence.

### Genomic annotation and analysis

Preliminarily gene annotation was performed using the online program Dual Organellar Genome Annotator (OGDRAW v1.2; Wyman et al., 2004) and cp Genome Annotation, Visualization, Analysis, and GenBank Submission Tool (Cheng et al., 2013) with plastid/bacterial genetic code and default conditions. Putative gene and protein sequences were BLAST-searched in non-redundant nucleotide database and non-redundant protein database to verify the exact gene and exon boundaries. All tRNA genes were further confirmed through online Trnascan-SE and tRNADB-CE search server (Griffiths-Jones et al., 2003; Schattner et al., 2005; Abe et al., 2011). The graphical map of the circular plastome was drawn using Organellar Genome DRAW (Lohse et al., 2007).

### Identification of simple-sequence repeats (SSRs)

The genomic sequence was applied to exploit potential SSRs by using MISA software (http://pgrc.ipk-gatersleben.de/misa/). Tandem repeats of 1–6 nucleotides were considered as microsatellites. The minimum numbers of repeats were set to 10, 6, 5, 5, 5, and 5 for mono-, di-, tri-, tetra-, penta-, and hexa-nucleotides, respectively.

### Long repeat analysis

Web-based REPuter (http://bibiserv.techfak.uni-bielefeld.de/reputer/) was used to analyze the repeat sequences, which included forward, reverse, and tandem repeats with minimal lengths of 30 bp and edit distances of less than 3 bp.

### Codon usage

Codon usage was determined for all protein-coding genes. Statistical analyses of the distributions and visualization of codon usage in the form of heatmaps of 28 species of *Angiosperms* and histogram were conducted using R language with relative synonymous codon usage (RSCU) value (Sharp and Li, 1987).

RSCU is a simple measure of non-uniform usage of synonymous codons in a coding sequence. The RSCU value is the number of times a particular codon is observed, relative to the number of times that the codon would be observed for a uniform synonymous codon usage (i.e., all codons for a given amino acid exhibit similar probabilities). The RSCU value in the absence of any codon usage bias is 1.00, which is the case for the CDS sequence in the following example. A codon used less frequently than expected will achieve RSCU of <1.00, whereas codons used more frequently than expected may reach RSCU of >1.00.

### Phylogenetic analysis

The jModeltest 0.1.1 software was employed to analyze the general GTR+G+I model for nucleotide sequence and HIVb+I+G model for protein sequence by using optimized parameters (Posada, 2008). Phylogenetic analysis was subsequently performed using Maximum likelihood (ML) and Bayesian inference (BI) methods. ML analysis was conducted using RAxML8.1.5 with 1000 bootstrap replicates (Stamatakis, 2014). BI analysis was conducted using Phylobayes 4.1b with two chain max diff <0.01 (Lartillot et al., 2009).

### CGView comparison tool (CCT) map

The *A. sinensis* cp genome was compared with other available cp genomes of Malvales by using CCT (Grant and Stothard, 2008). Genes were signed by Clusters of Orthologous Groups, and BLAST was used to align other genomes to *A. sinensis.* The results are shown as a circular map. AT distributions were measured on the basis of AT skewed using the equation: AT-skew = (A−T)/(A+T).

## Results and discussion

### Genome sequencing and assembly

**Figure 1:**
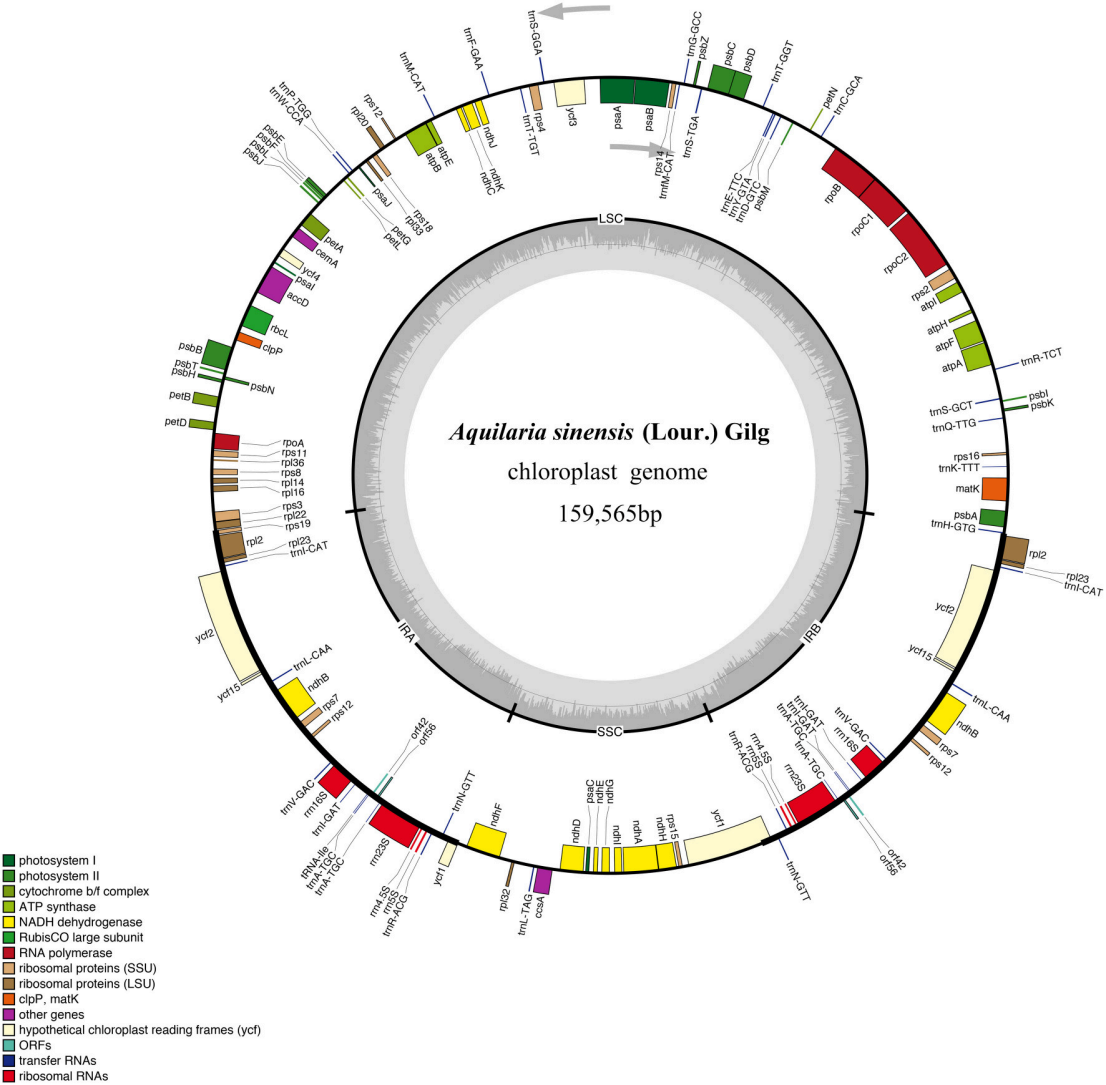
**Gene map of *A. sinensis* chloroplast (cp) genome sequence**. Organization of the cp genome of *A. sinensis* showing annotated genes. Genes drawn inside the circle are transcribed clockwise, and those outside are counter clockwise. Genes belonging to different functional groups are color-coded. The inner circle shows the locations of the large single-copy region, small single-copy, and the pair of inverted repeats (IRa and IRb). The darker gray in the inner circle corresponds to GC content, whereas the lighter gray corresponds to AT content.

A total of 2.48 × 10^7^ reads with an average read length of 125 bp were obtained after low-quality bases and adapter sequences were trimmed. *De novo* assembly produced 691,722 contigs (2.78%). The size of the *A. sinensis* cp genome was 159,565 bp (Figure 1). The genome included an LSC region of 87,482 bp, an SSC region of 19,857 bp, and a pair of IRs (IRa and IRb) of 26,113 bp each (Table 1). The GC content was 37.11% (Table S2). However, the GC content was unevenly distributed in the entire cp genome, with the highest value in the IR regions (42.86%), followed by the LSC (34.95%) and SSC (31.58%) regions. The frequency of codon usage was deduced for the cp genome on the basis of the CDS sequences. Notably, the AT contents were 54.64, 62.31, and 69.34% at the first, second, and third codon positions, respectively, within the protein-coding regions (Table S2). Bias toward higher AT content at the third codon position was consistent with the enrichment of A and T, which has been widely observed in many other sequenced land plant cp genomes (Morton, 1998; Tangphatsornruang et al., 2009; Nie et al., 2012; Qian et al., 2013). The sequences of the *A. sinensis* cp genome were deposited in GenBank with accession number KT148967.

**Table 1:** **Gene conten*ts in A. sinensis* chloroplast genome**.

**Category of genes**	**Group of gene**	**Name of gene**
Self-replication	Small subunit of ribosome	rps7^*^ rps11 rps15 rps14 rps19 rps18 rps4 rps3 rps2 rps8 rps12^*^ rps16
	Large subunit of ribosome	rpl23^*^ rpl20 rpl22 rpl2^*^ rpl36 rpl32 rpl33 rpl14 rpl16
	DNA-dependent RNA polymerase	rpoB rpoA rpoC2 rpoC1
	Ribosomal RNA genes	rrn16S^*^ rrn5S^*^ rrn23S^*^ rrn4.5S^*^
	Transfer RNA genes	trnQ-TTG trnR-ACG^*^ trnM-CAT trnY-GTA trnH-GTG trnA-TGC^*^ trnP-TGG
		trnS-GCT trnN-GTT^*^ trnL-TAG trnG-GCC trnW-CCA trnK-TTT
		trnT-TGT trnL-CAA^*^trnS-TGA trnI-CAT^*^ trnfM-CAT trnF-GAA trnS-GGA
		trnV-GAC^*^ trnC-GCA trnD-GTC trnE-TTC trnI-GAT^*^ trnT-GGT
Genes for photosynthesis	Subunits of NADH dehydrogenase	ndhB^*^ ndhC ndhA ndhF ndhG ndhD ndhE ndhJ ndhK ndhH ndhI
	Large subunit of Rubisco	rbcL
	Subunits of photosystem II	psbE psbD psbF psbA psbC psbB psbM psbL psbN psbI psbH psbK psbJ psbT psbZ
	Subunits of photosystem I	psaI psaJ psaB psaC ycf4 ycf3 psaA
	Subunits of ATP synthase	atpI atpH atpB atpA atpF atpE
	Subunits of cytochrome	petD petG petA petB petL petN
Other genes	Envelope membrane protein	cemA
	C-type cytochrome synthesis gene	ccsA
	Subunit of acetyl-CoA	accD
	Protease	clpP
	Maturase	matK
	Component of TIC complex	ycf1
Genes of unknown function	Conserved open reading frames	ycf2^*^ orf42 orf56
Pseudogenes		ycf1^*^ ycf15^*^

* *Duplicated gene*.

### Genomic annotation

The draft genome was drawn using OGDRAW v1.2 (Figure 1). The single collapsed IR contig was separated into two repeat regions. Assembly of the two IRs and LSC and SSC contigs covered the complete sequence without gaps. The positions of all genes identified in the cp genome and functional categorization of these genes are presented in Figure 1. The *A. sinensis* cp genome was 159,565 bp long with a typical quadripartite structure. A total of 113 functional genes were identified, which comprised 82 protein-coding genes, 27 tRNA genes, and 4 rRNA genes (Table 1). Comparing to the genes in other species (Figure S2), little change was found in gene structure. The very low level of recombination was also reported in the cp genome of land plant (Provan et al., 2001; Ravi et al., 2008). Among the 82 protein-coding genes, 75 were single-copy genes, and 7 were duplicates. Among the 31 RNA genes, 20 were unique, and 11 were duplicates. Among the 113 unique genes, 9 genes contained 1 intron (7 protein-coding and 2 tRNA genes), and 1 gene (*ycf3*) contained 2 introns (Table S3). The *ycf3* gene was similar to those in *Globe artichoke* and *Metasequoia glyptostroboides* (Chen et al., 2015; Curci et al., 2015). Out of the 10 genes with introns, 3 protein-coding genes were located in the LSC, 1 in the SSC, and 6 (4 protein-coding genes and 2 tRNAs) in the IR region. The *ndhA* gene presented the largest intron (1148 bp). In addition, *ndhB* and *rpl2* were identified as duplicate genes.

### SSR analysis

SSRs consist of 1–6 nucleotide repeat units, which are also known as microsatellites and short tandem repeats (Chen et al., 2006). SSRs are important in plant typing (Yang et al., 2011; Xue et al., 2012) and widely used for genetic molecular markers in population genetics (Doorduin et al., 2011; He et al., 2012). A total of 45 SSR regions were identified using the microsatellite identification tool (MISA) in *A. sinensis* cp genome (Table 2), accounting for 499 bp of the total sequence (0.3%), and 37 SSRs were only composed of A or T bases. Two SSRs were composed of C bases, and six SSRs were composed of dinucleotide (AT/TA/TC) repeats. Therefore, SSRs in *A. sinensis* cp genome were rich in AT. Poly(A)/(T) had been reported to exhibit higher proportion relative to poly(G)/(C) in many plant families (Kumar et al., 2009; Melotto-Passarin et al., 2011; Nie et al., 2012; Martin et al., 2013). Among these SSRs, 36 SSRs were located in noncoding sections of the LSC/SSC region, and 9 SSRs in protein-coding genes (*rpoC2, rpoB, psbF, cemA, psbN, rps19*, and *ycf1*). No tri- or tetra-nucleotide repeats over 15 bp long were found. The SSRs identified in this study may provide a new perspective to refine the phylogeny and elucidate the origin of cultivars.

**Table 2:** **Simple sequence repeats in *A. sinensis* chloroplast genome**.

**cpSSR ID**	**Repeat motif**	**Length (bp)**	**Start**	**End**	**Region**	**Annotation**
1	(A)10	10	1883	1892	LSC	
2	(T)10	10	2023	2032	LSC	
3	(A)12	12	4127	4138	LSC	
4	(A)12	12	4803	4814	LSC	
5	(C)10	10	5260	5269	LSC	
6	(A)10	10	6662	6671	LSC	
7	(A)15	15	7644	7658	LSC	
8	(A)11	11	8033	8043	LSC	
9	(T)11	11	8380	8390	LSC	
10	(T)11	11	9080	9090	LSC	
11	(T)10	10	9863	9872	LSC	
12	(TA)6	12	10743	10754	LSC	
13	(A)10	10	13927	13936	LSC	
14	(T)10	10	14100	14109	LSC	
15	(TC)7	14	17620	17633	LSC	
16	(T)11	11	18009	18019	LSC	rpoC2
17	(T)10	10	19917	19926	LSC	rpoC2
18	(T)10	10	27649	27658	LSC	rpoB
19	(T)10	10	30717	30726	LSC	
20	(A)10	10	32194	32203	LSC	
21	(T)10	10	44610	44619	LSC	
22	(A)10	10	45233	45242	LSC	
23	(A)10	10	47349	47358	LSC	
24	(A)11	11	47627	47637	LSC	
25	(T)10	10	48204	48213	LSC	
26	(T)12	12	51015	51026	LSC	
27	(T)11	11	51316	51326	LSC	
28	(T)10	10	51846	51855	LSC	
29	(A)11	11	61867	61877	LSC	
30	(T)11	11	62016	62026	LSC	
31	(A)11	11	62578	62588	LSC	
32	(TA)6	12	62964	62975	LSC	
33	(T)10	10	64002	64011	LSC	psbF
34	(C)10	10	66343	66352	LSC	
35	(T)12	12	67133	67144	LSC	cemA
36	(T)11	11	71791	71801	LSC	
37	(TA)6	12	77233	77244	LSC	psbN
38	(T)15	15	87280	87294	LSC	rps19
39	(TA)7	14	113802	113815	SSC	
40	(A)10	10	116349	116358	SSC	
41	(A)10	10	116494	116503	SSC	
42	(T)10	10	117050	117059	SSC	
43	(AT)7	14	117644	117657	SSC	
44	(T)10	10	129560	129569	SSC	ycf1^*^
45	(T)13	13	132293	132305	SSC	ycf1

* *Duplicated gene*.

### Large repeat analysis

Large repeat sequences showed repeats with length of ≥30 bp each. Sixty pairs of large repeat sequences with sequence identity of >90% were found in the *A. sinensis* cp genomes (Table 3). The repeats ranged from 30 to 600 bp in length and were repeated twice. A total of 33 large repeat sequences were located in protein-coding genes (e.g., *ycf1* and *ycf2*), and 27 large repeat sequences were located in the intergenic regions. Numerous repeated sequences were identified in cp genomes, particularly in the intergenic spacer regions, and have been reported in several angiosperm lineages (Yang et al., 2013).

**Table 3:** **Long repeat sequences in *A. sinensis* chloroplast genome**.

**id**	**Repeat Start 1**	**Type**	**Size(bp)**	**Repeat Start 2**	**Mismatch(bp)**	**E-value**	**Gene**	**Region**
1	1471	F	41	71846	−3	4.26E-10	IGS	LSC
2	1480	F	41	71855	−3	4.26E-10	IGS	LSC
3	1574	F	163	71953	−3	1.00E-81	IGS	LSC
4	1587	F	156	71966	−3	1.44E-77	IGS	LSC
5	8609	F	31	37664	−3	1.88E-04	IGS	LSC
6	41152	F	41	43376	−2	1.09E-11	psaB(CDS); psaA(CDS)	LSC
7	46415	F	36	101941	−1	1.64E-10	ycf3(intron); IGS	LSC; IRA
8	46415	F	36	124843	−3	2.92E-07	ycf3(intron); ndhA(inton)	LSC; SSC
9	46424	F	30	101950	−1	5.59E-07	ycf3(intron); IGS	LSC; IRA
10	70177	F	30	70366	−3	6.81E-04	accD(CDS)	LSC
11	70195	F	67	70273	−2	6.54E-27	accD(CDS)	LSC
12	70195	F	36	70291	−2	8.60E-09	accD(CDS)	LSC
13	70199	F	53	70259	−1	1.40E-20	accD(CDS)	LSC
14	70218	F	34	70260	−1	2.47E-09	accD(CDS)	LSC
15	70260	F	31	70296	−1	1.44E-07	accD(CDS)	LSC
16	95892	F	31	95928	−3	1.88E-04	ycf2(CDS)	IRA
17	96540	F	31	150227	−3	1.88E-04	ycf15(CDS)	IRA; IRB
18	97906	F	30	148863	−2	2.43E-05	IGS	IRA; IRB
19	101938	F	41	124840	−2	1.09E-11	IGS; ndhA(intron)	IRA; SSC
20	112678	F	31	112720	0	1.55E-09	IGS	IRA
21	113385	F	55	113454	0	5.52E-24	IGS	IRA
22	118510	F	64	118590	−1	4.04E-27	ccsA(CDS)	SSC
23	119996	F	32	120064	−3	5.20E-05	IGS	SSC
24	131641	F	30	131707	−3	6.81E-04	ycf1(CDS)	SSC
25	132792	F	31	132831	−2	6.50E-06	ycf1(CDS)	SSC
26	133153	F	39	133216	−3	5.85E-09	ycf1(CDS)	SSC
27	133168	F	38	133231	−1	1.08E-11	ycf1(CDS)	SSC
28	133282	F	62	133351	0	3.37E-28	ycf1(CDS)	SSC; IRB
29	150839	F	31	150875	−3	1.88E-04	ycf2(CDS)	IRB
30	8610	I	30	47858	−1	5.59E-07	IGS	LSC
31	37665	I	30	47858	−3	6.81E-04	IGS	LSC
32	46415	I	36	144821	−1	1.64E-10	ycf3(intron); IGS	LSC; IRB
33	46424	I	30	144818	−1	5.59E-07	ycf1(CDS)	LSC; IRB
34	89862	I	30	156882	−3	6.81E-04	ycf2(CDS)	IRA; IRB
35	89886	I	30	156906	−3	6.81E-04	ycf2(CDS)	IRA; IRB
36	94593	I	38	152149	−2	6.00E-10	ycf2(CDS)	IRA; IRB
37	94611	I	38	152167	−2	6.00E-10	ycf2(CDS)	IRA; IRB
38	95892	I	31	150839	−3	1.88E-04	ycf2(CDS)	IRA; IRB
39	95900	I	41	150839	0	1.48E-15	ycf2(CDS)	IRA; IRB
40	95918	I	41	150857	0	1.48E-15	ycf2(CDS)	IRA; IRB
41	95928	I	31	150875	−3	1.88E-04	ycf2(CDS)	IRA; IRB
42	112668	I	41	134047	−1	1.82E-13	IGS	IRA; IRB
43	112668	I	62	134047	−2	5.73E-24	IGS; ycf1(CDS),IGS	IRA; IRB
44	112678	I	31	134047	0	1.55E-09	IGS	IRA; IRB
45	112689	I	62	134068	−2	5.73E-24	IGS	IRA; IRB
46	112699	I	31	134047	−1	1.44E-07	IGS	IRA; IRB
47	112710	I	41	134089	−1	1.82E-13	IGS	IRA; IRB
48	112720	I	31	134089	0	1.55E-09	IGS	IRA; IRB
49	112720	I	31	134068	−1	1.44E-07	IGS	IRA; IRB
50	113385	I	70	133274	−1	1.08E-30	IGS; ycf1(CDS)	IRA
51	113454	I	55	133358	0	5.52E-24	IGS; ycf1(CDS)	IRA; SSC
52	113526	I	530	117403	0	0.00E+00	IGS,rpl(CDS)	IRA; SSC
53	124840	I	41	144819	−2	1.09E−11	ndhA(CDS,intron); IGS	SSC; IRB
54	59836	T	31	59866	0	1.55E-09	rps18	LSC
55	112668	T	41	112710	−1	1.82E−13	IGS	IRA
56	118496	T	78	118576	−3	1.61E−31	IGS	SSC
57	132782	T	41	132821	−3	4.26E−10	ycf1(CDS)	SSC
58	133193	T	44	133235	0	2.31E−17	ycf1(CDS)	SSC
59	133274	T	70	133343	−1	1.08E-30	ycf1(CDS)	SSC; IRB
60	134047	T	41	134089	−1	1.82E-13	IGS	IRB

F, Forward; I, Inverted; T, Tandem; IGS, intergenic space.

### Codon usage

Most protein-coding genes in these basal eudicots employ the standard ATG as the initiator codon. However, ATA, ATC, TTG, and ATT are also used as alternatives to ATG as the start codon. Among the *A. sinensis* cp protein-coding genes, nine genes were used alternatively to ATG as the start codon as follows: ATA for *atpF*; ATT for *ycf1* and *petB*; ATC for *rpl16*; GTG for *rps8, psbC*, and *ndhD*; and TTG for *ndhA* and *rpoC1.* In the *N. tabacum* cp genome, GTG was used as start codon for *rps19, psbC*, and *ycf15*, whereas ACG was used for *psbL* and *ndhD* (Sugiura et al., 1998). ACG and GTG were used as start codon for *rpl2* and *rps19*, as reported in *Oryza sativa* (Liu and Xue, 2004).

Furthermore, the codon usage patterns of the 82 distinct cp protein-coding genes in *A. sinensis* were examined. All the protein-coding genes were composed of 26,160 codons. Interestingly, as synonymous codons, almost each of these codons contained half synonymous codon, which ended with A or T with high RSCU values, and the other half ended with C or G with low RSCU values (Table S4). These codon usage patterns may be driven by the composition bias of the high proportion of A/T similar to those of other reported cp genomes (Raubeson et al., 2007; Delannoy et al., 2011) and mitochondrial genomes (Barth and Berendonk, 2011).

**Figure 2:**
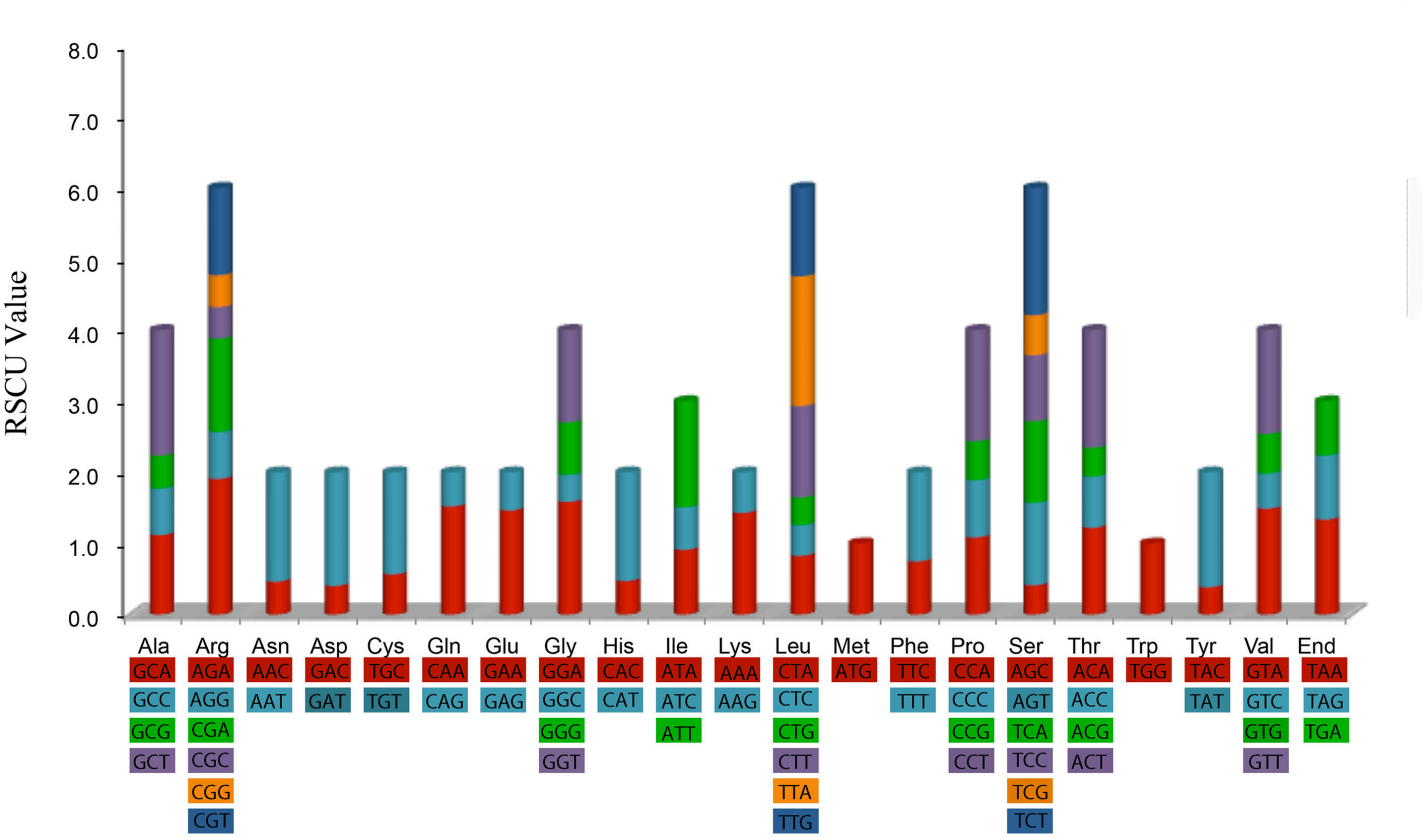
**Codon content of 20 amino acid and stop codon of 82 coding genes of *Aquilaria sinensis* chloroplast genome**. Color of the histogram is corresponding to the color of codons.

Figure 2 shows that the RSCU value increased with the number of codons that code a particular amino acid. The high RSCU value was probably attributed to the function of the amino acid or the structure of the peptide to avoid error in transcription.

**Figure 3:**
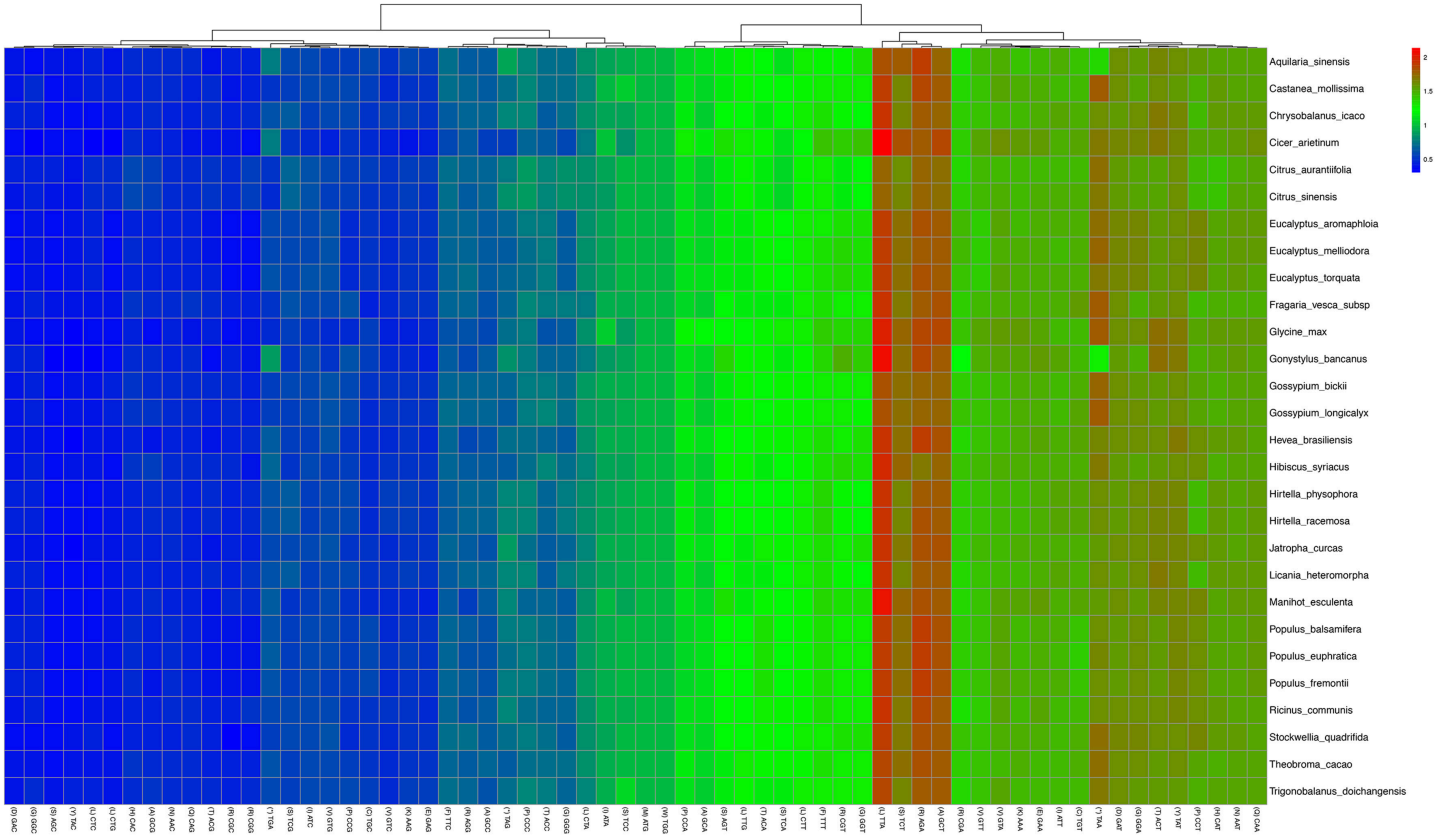
**Codon distribution of all merged protein-coding genes for all considered species**. Color key: higher red values indicate higher RSCU value and lower blue values indicate lower RSCU value; A Hierarchical clustering (Lance and Williams, 1967; average linkage method) was performed for codon patterns (x-axis).

Statistical analyses of the distributions and visualization of codon usage in the form of heatmaps of 28 species of *Angiosperms* (Figure 3) showed that approximately half of the codons were not frequently used. These codons were denoted in blue, which indicated RSCU value of <1 and weak codon bias. Almost two-thirds of all the codons with high RSCU values ended with purine (A/T). Thus, we hypothesized that the codon in *A. sinensis* cp genome bias ended with A/T. This phenomenon was also found in many other plant and algal lineages (Morton, 1998). The distribution of codon usage *in A. sinensis* cp genome was most similar to the codon usage in *Gonystylus bancanus* cp genome.

### Phylogenetic analyses

**Figure 4:**
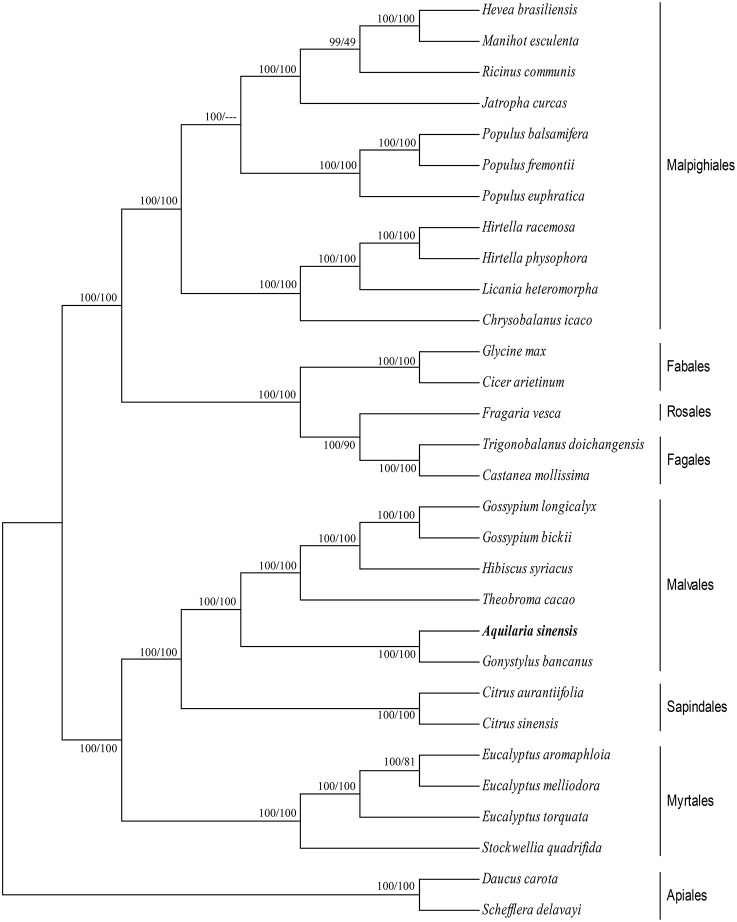
**Phylogenetic position of *A.sinensis* inferred by Maximum likelihood (ML) and Bayesian inference (BI) nucleic acid analyses of 82 protein-coding genes**. The first number above the lines indicates the BI bootstrap value of the nucleic acid analysis for each clade, whereas the second number indicates the ML bootstrap value. The position of *A.sinensis*is shown in boldface.

**Figure 5:**
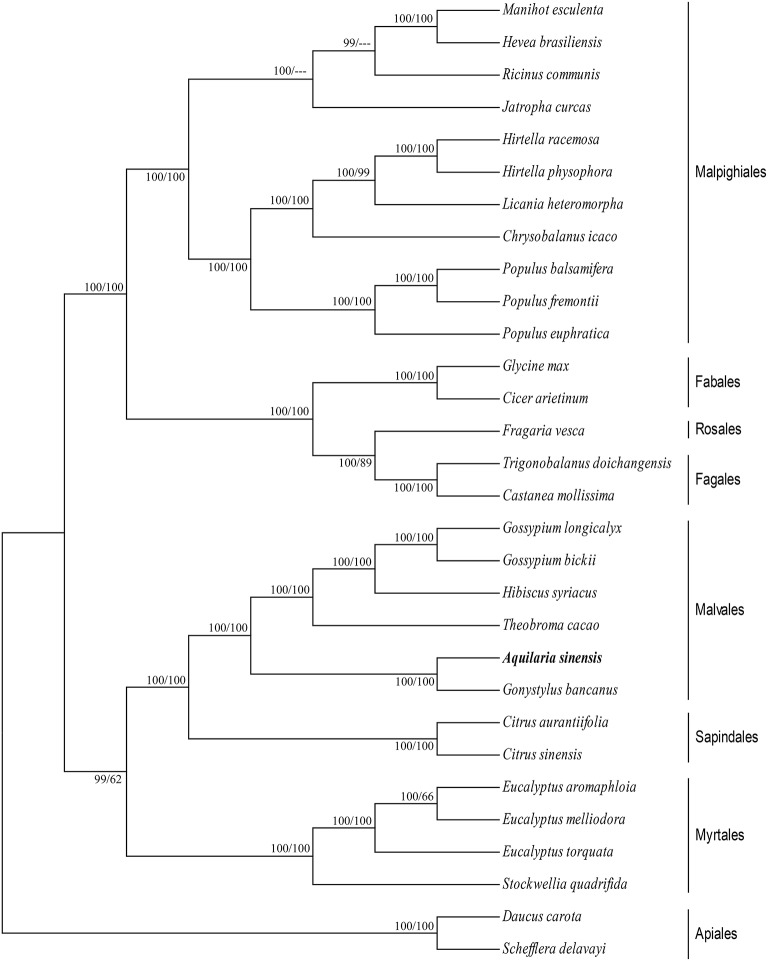
**Phylogenetic position of *A.sinensis* inferred by ML and BI protein analyses of 82 protein-coding genes**. The first number above the lines indicates the BI bootstrap value of the protein analysis for each clade, whereas the second number indicates the ML bootstrap value. The position of *A.sinensis* is shown in boldface.

cp genomes are significant in the research of phylogenetics, evolution, and molecular systematics. Numerous analytical studies have been conducted in the past decade to address phylogenetic issues at deep nodes by comparing multiple protein-coding genes (Lemieux et al., 2000; Delas Rivas et al., 2002; Moore et al., 2010) and complete sequences in cp genomes (Goremykin et al., 2004; Moore et al., 2007). These studies enhanced the understanding about enigmatic evolutionary relationships among *Angiosperms*. We examined the phylogenetic position of *A. sinensis* and relationships within *Angiosperms.* We previously selected 82 protein-coding genes commonly present in cp genomes of 29 species, including the *A. sinensis* cp genome sequenced in the current study. ML and BI nucleic acid analyses were performed, and the results are summarized in Figures 4, 5. Similar phylogenetic topologies were found in the ML and BI nucleic acid analyses. Bootstrap values were very high, and 28 of 29 nodes with 100% bootstrap values were found using ML. Up to 25 out of the 29 nodes with bootstrap values of ≥99% were found using BI. *A. sinensis* and *G. bancanus* were grouped in Malvales, with 100% bootstrap values in the ML and BI phylogenetic trees. Similarly, in the ML protein analyses, 27 of the 29 nodes yielded bootstrap values of 100%, and 24 nodes reached ≥99% in the BI. ML and BI protein analyses showed that *A. sinensis* can also be grouped with *G. bancanus* within Malvales. The phylogenetic results strongly support the position of *A.sinensis* within the Malvales order. However, the results were inconsistent because *A.sinensis* was classified into *Myrtales* according to the traditional morphological classification of China.

### CCT map

**Figure 6:**
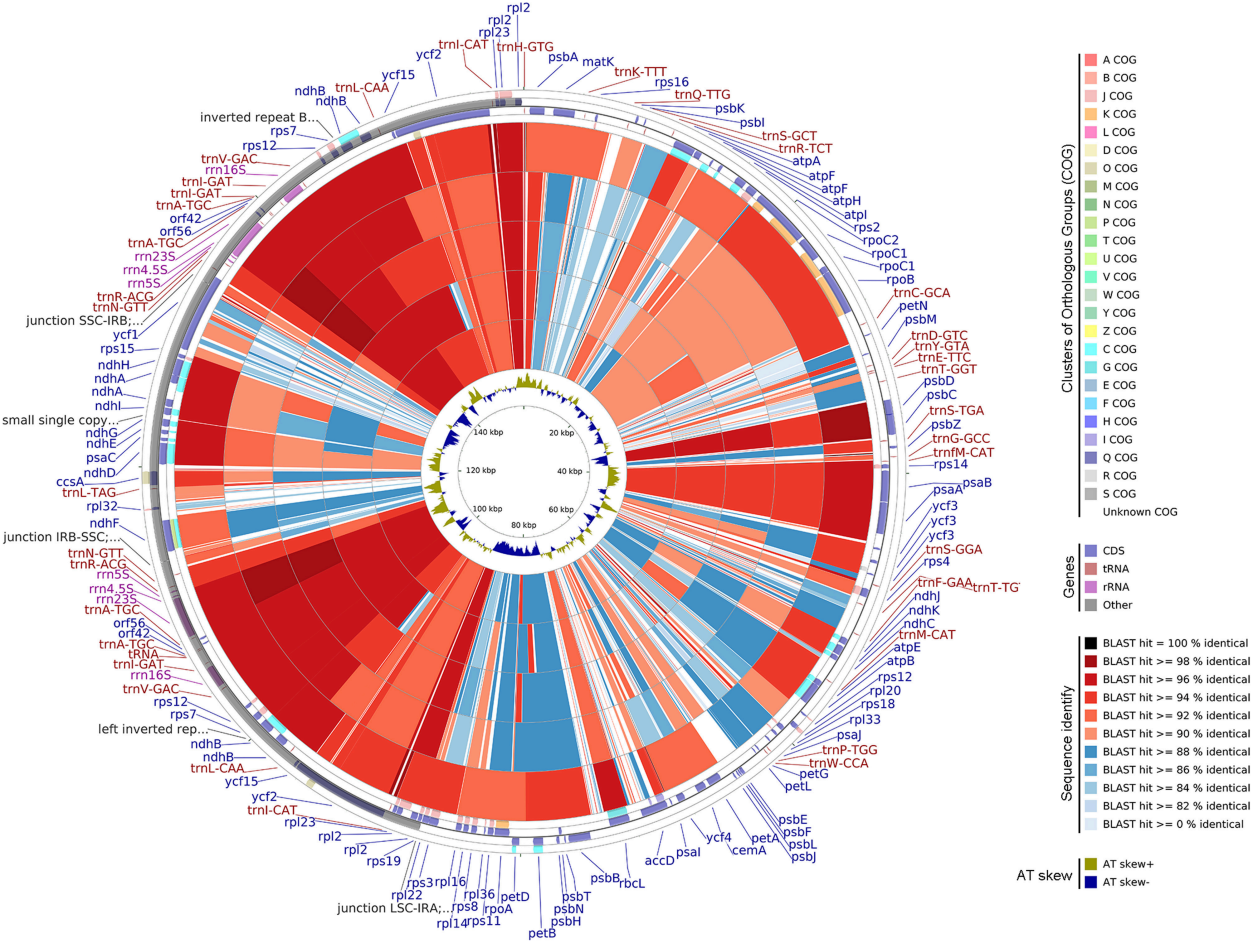
**Genome comparison of five CP genomes of Malvales to *A.sinensis***. From the outer to the inner color ring: *Gonystylus bancanus, Theobroma cacao, Gossypium longicalyx, Hibiscus syriacus*, and *Gossypium bickii*. BLAST was used to align other genomes to *A. sinensis*, and the results are shown with a circular map. The color codes are based on the similarity score, that is, dark red and blue depict similarity scores of 100%, above 90%, and below 90%, respectively. The four outer narrow rings are the protein-coding gene positions based on the *A. sinensis* cp genome. The color codes are based on Clusters of Orthologous Groups. The innermost ring is AT skew in the *A. sinensis.* AT skew+ indicate A>T, AT skew- indicate A < T.

Five available cp genomes of Malvales were compared with the *A.sinensis* cp genome by using CCT (Figure 6). The sequence identity between the *A.sinensis* cp and other representatives of the Malvales was analyzed. The results showed that *G. bancanus* cp genome achieved the highest sequence similarity (>90%), which was consistent with the result of the phylogenetic analysis. The highest similar region across all five cp genomes occurred in the IR region. LSC and SSC regions were less conservative; thus, many regions with identity results were lower than 90%, such as the *ycf1* gene in the SSC region and intergenic space regions. This evolutionary conserved feature was reported in the IR region in cp (Curtis and Clegg, 1984; Palmer, 1985).

## Conclusions

The complete cp sequence of traditional medicinal plant *A. sinensis* was assembled, annotated, and analyzed. The cp genome consisted of one LSC, one SSC, and two IR regions. A total of 45 polymorphic SSR loci and 60 pairs of large repeats were identified in the *A. sinensis* cp genome. These repeat motifs can be selected to develop markers and conduct phylogenetic analysis. Both ML and BI phylogenetic analyses strongly supported the position of *A. sinensis* as a sister to *G. bancanus* within the Malvales order. The distribution of codon usage in *A. sinensis* cp genome was most similar to that in *G. bancanus* cp genome. CCT analytical results also indicated that the *A. sinensis* cp genome achieved higher sequence similarity than the *G. bancanus* cp genome. However, the traditional morphological classification of China classified *A. sinensis* into *Myrtales*. The data obtained in this study will be beneficial for further investigations on *A. sinensis*. Moreover, the results will help expand the understanding about the evolutionary history of the Malvales order, particularly regarding the role of *A. sinensis* in plant systematics and evolution.

## Author contributions

YW designed and performed the experiment as well as drafted the manuscript. XC and SP conceived the study and revised the manuscript. DZ, XJ, WM, and HD prepared the samples and analyzed part of the data. All the authors have read and approved the final manuscript.

### Conflict of interest statement

The authors declare that the research was conducted in the absence of any commercial or financial relationships that could be construed as a potential conflict of interest.

## Supplementary material

Figure S1 **Coverage depth of contigs of *A. sinensis* CP genome**.

Figure S2 **Comparisons of CP genomic structure among five land plants, including *A. sinensis*, using Easyfig**. The arrowheads indicate genes with their orientations and positions. The orange arrows indicate the coding regions of genes. Gray ligature indicate positions and similarity of the genes of five land plants.

Table S1 **List of CP genomes used as reference to map to contigs**.

Table S2 **Summary of *A. sinensis* chloroplast genome characteristics**.

Table S3 **The genes in the *A. sinensis* chloroplast genome with introns**.

Table S4 **Relative synonymous codon usage (RSCU) for the *A. sinensis* chloroplast genome**.

## References

[B1] Abe T. Ikemura T. Sugahara J. Kanai A. Ohara Y. Uehara H.. (2011). tRNADB-CE 2011: tRNA gene database curated manually by experts. Nucleic Acids Res. 39, 210–213. 10.1093/nar/gkq1007 21071414 PMC3013639

[B2] Awasthi P. Ahmad I. Gandhi S. G. Bedi Y. S. (2012). Development of chloroplast microsatellite markers for phylogenetic analysis in Brassicaceae. Acta Biol. Hung. 63, 463–473. 10.1556/ABiol.63.2012.4.5 23134603

[B3] Barth D. Berendonk T. U. (2011). The mitochondrial genome sequence of the ciliate Paramecium caudatum reveals a shift in nucleotide composition and codon usage within the genus Paramecium. BMC Genomics 12:272. 10.1186/1471-2164-12-272 21627782 PMC3118789

[B4] Bedbrook J. R. Bogorad L. (1976). Endonuclease recognition sites mapped on Zea mays chloroplast DNA. Proc. Natl. Acad. Sci. U.S.A. 73, 4309–4313. 10.1073/pnas.73.12.4309 16592373 PMC431441

[B5] Chen C. Zhou P. Choi Y. A. Huang S. Gmitter F. G. Jr. (2006). Mining and characterizing microsatellites from citrus ESTs. Theor. Appl. Genet. 112, 1248–1257. 10.1007/s00122-006-0226-1 16474971

[B6] Chen H. Melis A. (2013). Marker-free genetic engineering of the chloroplast in the green microalga *Chlamydomonas reinhardtii*. Plant Biotechnol. J. 11, 818–828. 10.1111/pbi.12073 23647698

[B7] Chen J. H. Hao Z. D. Xu H. B. Yang L. M. Liu G. X. Sheng Y. (2015). The complete chloroplast genome sequence of the relict woody plant *Metasequoia glyptostroboides* Huet Cheng. Front. Plant. Sci. 6:447 10.3389/fpls.2015.00447 26136762 PMC4468836

[B8] Cheng J. Zeng X. Ren G. Liu Z. (2013). CGAP: a new comprehensive platform for the comparative analysis of chloroplast genomes. BMC Bioinformatics 14:95. 10.1186/1471-2105-14-95 23496817 PMC3636126

[B9] China pharmacopoeia Committee (2010). The Pharmacopoeia of People's Republic of China. Beijing: Chemical Industry Press.

[B10] Ching P. L. Jen P. H. Chung S. Wu Chih, Y. H. Shu M. C. (2010). Comparative chloroplast genomics reveals the evolution of *Pinaceae Genera* and subfamilies. Genome Biol. Evol. 2, 504–517. 10.1093/gbe/evq036 20651328 PMC2997556

[B11] Corriveau J. L. Coleman A. W. (1988). Rapid screening method to detect potential biparental inheritance of plastid DNA and results for over 200 angiosperm species. Am. J. Bot. 75, 1443–1458. 10.2307/2444695

[B12] Curci P. L. De Paola D. Danzi D. Vendramin G. G. Sonnante G. (2015). Complete Chloroplast genome of the multifunctional crop *Globe Artichoke* and comparison with other *Asteraceae*. PLoS ONE 10:e0120589. 10.1371/journal.pone.0120589 25774672 PMC4361619

[B13] Curtis S. E. Clegg M. T. (1984). Molecular evolution of chloroplast DNA sequences. Mol. Biol. Evol. 1, 291–301. 6152869 10.1093/oxfordjournals.molbev.a040319

[B14] Delannoy E. Fujii S. Des Francs-Small C. C. Brundrett M. Small I. (2011). Rampant gene loss in the underground orchid *Rhizanthella gardneri* highlights evolutionary constraints on plastid genomes. Mol. Biol. Evol. 28, 2077–2086. 10.1093/molbev/msr028 21289370 PMC3112369

[B15] Delas Rivas J. Lozano J. J. Ortiz A. R. (2002). Comparative analysis of chloroplast genomes: functional annotation, genome-based phylogeny, and deduced evolutionary patterns. Genome Res. 12, 567–583. 10.1101/gr.209402 11932241 PMC187516

[B16] Doorduin L. Gravendeel B. Lammers Y. Ariyurek Y. Chin-A-Woeng T. Vrieling K. (2011). The complete chloroplast genome of 17 individuals of pest species *Jacobaeavulgaris*: SNPs,microsatellites and barcoding markers for population and phylogenetic studies. DNA Res. 18, 93–105. 10.1093/dnares/dsr002 21444340 PMC3077038

[B17] Gaudeul M. Gardner M. F. Thomas P. Ennos R. A. Hollingsworth P. M. (2014). Evolutionary dynamics of emblematic *Araucaria* species (Araucariaceae) in New Caledonia: nuclear and chloroplast markers suggest recent diversification, introgression, and a tight link between genetics and geography within species. BMC Evol. Biol. 14:171. 10.1186/s12862-014-0171-6 25189104 PMC4182765

[B18] Goremykin V. V. Hirsch-Ernst K. I. Wolfl S. Hellwig F. H. (2004). The chloroplast genome of Nymphaea alba: whole-genome analyses and the problem of identifying the most basal angiosperm. Mol. Biol. Evol. 21, 1445–1454. 10.1093/molbev/msh147 15084683

[B19] Grant J. R. Stothard P. (2008). The CGView Server: a comparative genomics tool for circular genomes. Nucleic. Acids. Res. 36 (Web Server issue), W181–W184. 10.1093/nar/gkn179 18411202 PMC2447734

[B20] Griffiths-Jones S. Bateman A. Marshall M. Khanna A. Eddy S. R. (2003). Rfam: an RNA family database. Nucleic Acids Res. 31, 439–441. 10.1093/nar/gkg006 12520045 PMC165453

[B21] He S. Wang Y. Volis S. Li D. Yi T. (2012). Genetic diversity and population structure: implications for conservation of wildsoybean (*Glycinesoja* Sieb.et Zucc)based on nuclear and chloroplast microsatellite variation. Int. J. Mol. Sci. 13, 12608–12628. 10.3390/ijms131012608 23202917 PMC3497291

[B22] Jheng C. Chen T. Lin J. Chen T. Wu W. Chang C. (2012). The comparative chloroplast genomic analysis of photosynthetic orchids and developing DNA markers to distinguish *Phalaenopsis* orchids. Plant Sci. 190, 62–73. 10.1016/j.plantsci.2012.04.001 22608520

[B23] Kumano M. (1986). Clone bank of the tobacco (*Nicotiana tabacum*) chloroplast genome as a set of overlapping restriction endonuclease fragments: mapping of eleven ribosomal protein genes. Plant Sci. 44, 211–216. 10.1016/0168-9452(86)90093-2

[B24] Kumar S. Hahn F. M. McMahan C. M. Cornish K. Whalen M. C. (2009). Comparative analysis of the complete sequence of the plastid genome of *Parthenium argentatum* and identification of DNA barcodes to differentiate Parthenium species and lines. BMC Plant Biol. 9:131. 10.1186/1471-2229-9-131 19917140 PMC2784773

[B25] Lance G. N. Williams W. T. (1967). A general theory of classificatory sorting strategies 1. hierarchical systems. Comput. J. 9, 373–380. 10.1093/comjnl/9.4.373

[B26] Lartillot N. Lepage T. Blanquart S. (2009). PhyloBayes 3: a Bayesian software package for phylogenetic reconstruction and molecular dating. Bioinformatics. 25, 2286–2288. 10.1093/bioinformatics/btp368 19535536

[B27] Lemieux C. Otis C. Turmel M. (2000). Ancestral chloroplast genome in Mesostigma viride reveals an early branch of green plant evolution. Nature 403, 649–652. 10.1038/35001059 10688199

[B28] Li H. Durbin R. (2009). Fast and accurate short read alignment with Burrows-Wheeler transform. Bioinformatics 25, 1754–1760. 10.1093/bioinformatics/btp324 19451168 PMC2705234

[B29] Liu Q. B. Xue Q. Z. (2004). Codon usage in the chloroplast genome of rice (*Oryza sativa* L. ssp. japonica). Acta Agron. Sin. 30, 1220–1224.

[B30] Lohse M. Drechsel O. Bock R. (2007). Organellar Genome DRAW (OGDRAW): a tool for the easy generation of high-quality custom graphical maps of plastid and mitochondrial genomes. Curr. Genet. 52, 267–274. 10.1007/s00294-007-0161-y 17957369

[B31] Luo R. Liu B. Xie Y. Li Z. Huang W. Yuan J.. (2012). SOAPdenovo2: an empirically improved memory-efficient short-read *de novo* assembler. Gigascience 1:18. 10.1186/2047-217x-1-18 23587118 PMC3626529

[B32] Martin G. Baurens F. C. Cardi C. Aury J. M. D'Hont A. (2013). The complete chloroplast genome of banana (*Musa acuminata*, Zingiberales): insight into plastid monocotyledon evolution. PLoS ONE 8:e67350. 10.1371/journal.pone.0067350 23840670 PMC3696114

[B33] Melotto-Passarin D. M. Tambarussi E. V. Dressano K. De Martin V. F. Carrer H. (2011). Characterization of chloroplast DNA microsatellites from Saccharum spp and related species. Genet. Mol. Res. 10, 2024–2033. 10.4238/vol10-3gmr1019 21948764

[B34] Moore M. J. Bell C. D. Soltis P. S. Soltis D. E. (2007). Using plastid genome-scale data to resolve enigmatic relationships among basal angiosperms. Proc. Natl. Acad. Sci. U.S.A. 104, 19363–19368. 10.1073/pnas.0708072104 18048334 PMC2148295

[B35] Moore M. J. Soltis P. S. Bell C. D. Burleigh J. G. Soltis D. E. (2010). Phylogenetic analysis of 83 plastid genes further resolves the early diversification of eudicots. Proc. Natl. Acad. Sci. U.S.A. 107, 4623–4628. 10.1073/pnas.0907801107 20176954 PMC2842043

[B36] Morton B. R. (1998). Selection on the codon bias of chloroplast and cyanelle genes indifferent plant and algal lineages. J. Mol. Evol. 46, 449–459. 10.1007/PL00006325 9541540

[B37] Neuhaus H. E. Emes M. J. (2000). Nonphoto synthetic metabolism in plastids. Annu. Rev. Plant Physiol. Plant Mol. Biol. 51, 111–140. 10.1146/annurev.arplant.51.1.111 15012188

[B38] Nie X. Lv S. Zhang Y. Du X. Wang L. Biradar S. S.. (2012). Complete chloroplast genome sequence of a major invasive species, crofton weed (*Ageratinaadenophora*). PLoS ONE 7:e36869. 10.1371/journal.pone.0036869 22606302 PMC3350484

[B39] Palmer J. D. (1985). Comparative organization of chloroplast genomes. Annu. Rev. Genet. 19, 325–354. 10.1146/annurev.ge.19.120185.001545 3936406

[B40] Palmer J. D. (1991). Plastid chromosomes: structure and evolution, in Molecular Biology of Plastids, ed Bogorad L. (San Diego, CA: Academic Press), 5–53.

[B41] Posada D. (2008). jModelTest: phylogenetic model averaging. Mol. Biol. Evol. 25, 1253–1256. 10.1093/molbev/msn083 18397919

[B42] Provan J. Powell W. Hollingsworth P. M. (2001). Chloroplast microsatellites: new tools for studies in plant ecology and evolution. Trends Ecol. Evol. 16, 142–147. 10.1016/S0169-5347(00)02097-8 11179578

[B43] Qian J. Song J. Gao H. Zhu Y. Xu J. Pang X.. (2013). The complete chloroplast genome sequence of the medicinal plant *Salvia miltiorrhiza*. PLoS ONE 8:e57607. 10.1371/journal.pone.0057607 23460883 PMC3584094

[B44] Raubeson L. A. Jansen R. K. (2005). Chloroplast genomes of plants, in Plant Diversity and Evolution: Genotypic and Phenotypic Variation in Higher Plants, ed Henry R. J. (Cambridge, MA: CABI), 45–68.

[B45] Raubeson L. A. Peery R. Chumley T. W. Dziubek C. Fourcade H. M. Boore J. L.. (2007). Comparative chloroplast genomics: analyses including new sequences from the angiosperms *Nuphar advena* and *Ranunculus macranthus*. BMC Genomics 8:174. 10.1186/1471-2164-8-174 17573971 PMC1925096

[B46] Ravi V. Khurana J. P. Tyagi A. K. Khurana P. (2008). An update on chloroplast genome. Plant Syst. Evol. 271, 101–122. 10.1007/s00606-007-0608-0

[B47] Sato S. Nakamura Y. Kaneko T. Asamizu E. Tabata S. (1999). Complete structure of the chloroplast genome of *Arabidopsis thaliana*. DNAquilaria Res. 6, 283–290. 10.1093/dnares/6.5.283 10574454

[B48] Schattner P. Brooks A. N. Lowe T. M. (2005). The tRNAscan-SE, snoscan and snoGPS web servers for the detection of tRNAs and snoRNAs. Nucleic Acids Res. 33, W686–W689. 10.1093/nar/gki366 15980563 PMC1160127

[B49] Sharp P. M. Li W. H. (1987). The codon adaptation index–a measure of directional synonymous codon usage bias, and its potential applications. Nucleic Acids Res. 15, 1281–1295. 10.1093/nar/15.3.1281 3547335 PMC340524

[B50] Stamatakis A. (2014). RAxML Version 8: a tool for phylogenetic analysis and post-analysis of large phylogenies. Bioinformatics 30, 1312–1313. 10.1093/bioinformatics/btu033 24451623 PMC3998144

[B51] Steele P. R. Hertweck K. L. Mayfield D. McKain M. R. Leebens-Mack J. Pires J. C.. (2012). Quality and quantity of data recovered from massively parallel sequencing: examples in asparagales and poaceae. Am. J. Bot. 99, 330–348. 10.3732/ajb.1100491 22291168

[B52] Straub S. C. Parks M. Weitemier K. Fishbein M. Cronn R. C. Liston A. (2012). Navigating the tip of the genomic iceberg: next-generation sequencing for plant systematics. Am. J. Bot. 99, 349–364. 10.3732/ajb.1100335 22174336

[B53] Sugiura M. Hirose T. Sugita M. (1998). Evolution and mechanism of translation in chloroplast. Annu. Rev. Genet. 32, 437–459. 10.1146/annurev.genet.32.1.437 9928487

[B54] Takano A. Okada H. (2011). Phylogenetic relationships among subgenera, species, and varieties of Japanese *Salvia* L. (Lamiaceae). J. Plant. Res. 124, 245–252. 10.1007/s10265-010-0367-9 20628783

[B55] Tangphatsornruang S. Sangsrakru D. Chanprasert J. Uthaipaisanwong P. Yoocha T. Jomchai N.. (2009). The chloroplast genome sequence of mungbean (*Vignaradiata*) determined by high-throughput pyrosequencing: structural organization and phylogenetic relationships. DNAquilaria Res. 17, 1–22. 10.1093/dnares/dsp025 20007682 PMC2818187

[B56] Turner B. Munzinger J. E. R. O. Duangjai S. Temsch E. M. Stockenhuber R. Barfuss M. H.. (2013). Molecular phylogenetics of New Caledonian *Diospyros* (Ebenaceae) using plastid and nuclear markers. Mol. Phylogenet. Evol. 69, 740–763. 10.1016/j.ympev.2013.07.002 23850609 PMC3913082

[B57] Wicke S. Muller K. F. de Pamphilism C. W. Quandt D. Wickett N. J. Zhang Y.. (2013). Mechanisms of functional and physical genome reduction in photosynthetic and nonphotosynthetic parasitic plants of the broomrape family. Plant Cell. 25, 3711–3725. 10.1105/tpc.113.113373 24143802 PMC3877813

[B58] Wolfe K. H. Li W. H. Sharp P. M. (1987). Rates of nucleotide substitution vary greatly among plant mitochondrial, chloroplast, and nuclear DNAs. Proc. Natl. Acad. Sci. U.S.A. 84, 9054–9058. 10.1073/pnas.84.24.9054 3480529 PMC299690

[B59] Wyman S. K. Jansen R. K. Boore J. L. (2004). Automatic annotation of organellar genomes with DOGMA. Bioinformatics 20, 3252–3255. 10.1093/bioinformatics/bth352 15180927

[B60] Xue J. Wang S. Zhou S. L. (2012). Polymorphic chloroplast microsatel- lite loci in Nelumbo (Nelumbonaceae). Am. J. Bot. 99, e240–e244. 10.3732/ajb.1100547 22615305

[B61] Yang A. H. Zhang J. J. Yao X. H. Huang H. W. (2011). Chloroplast microsatellite markers in *Liriodendron tulipifera* (Magnoliaceae) and cross- species amplification in L.chinense. Am. J. Bot. 98, e123–e126. 10.3732/ajb.1000532 21613178

[B62] Yang J. B. Yang S. X. Li H. T. Yang J. Li D. Z. (2013). Comparative chloroplast genomes of Camellia species. PLoS ONE 8:e73053. 10.1371/journal.pone.0073053 24009730 PMC3751842

[B63] Zhang Q. Liu Y. Sodmergen (2003). Examination of the cytoplasmic DNA in male reproductive cells to determine the potential for cytoplasmic inheritance in 295 angiosperm species. Plant Cell Physiol. 44, 941–951. 10.1093/pcp/pcg121 14519776

