# Supplementary figures and images for: Complete Chloroplast Genome Sequence of Aquilaria sinensis (Lour.) Gilg and Evolution Analysis within the Malvales Order
Source: Front Plant Sci. 2016 Mar 8;7:280. doi: 10.3389/fpls.2016.00280 (PMC4781844; doi:10.3389/fpls.2016.00280)

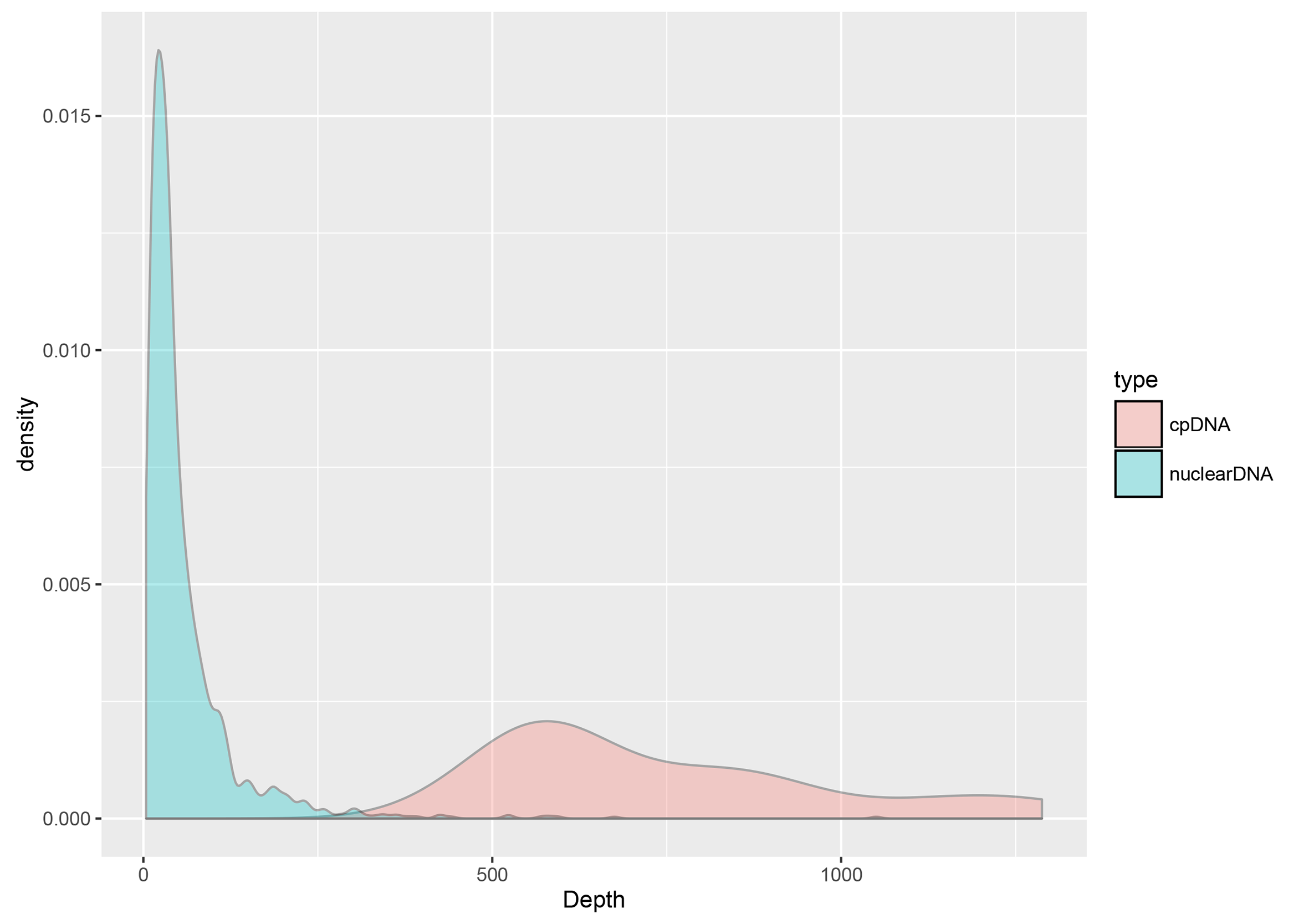

Supplement: Figure S1 — Coverage depth of contigs of A. sinensis CP genome. [file Image1.TIF]

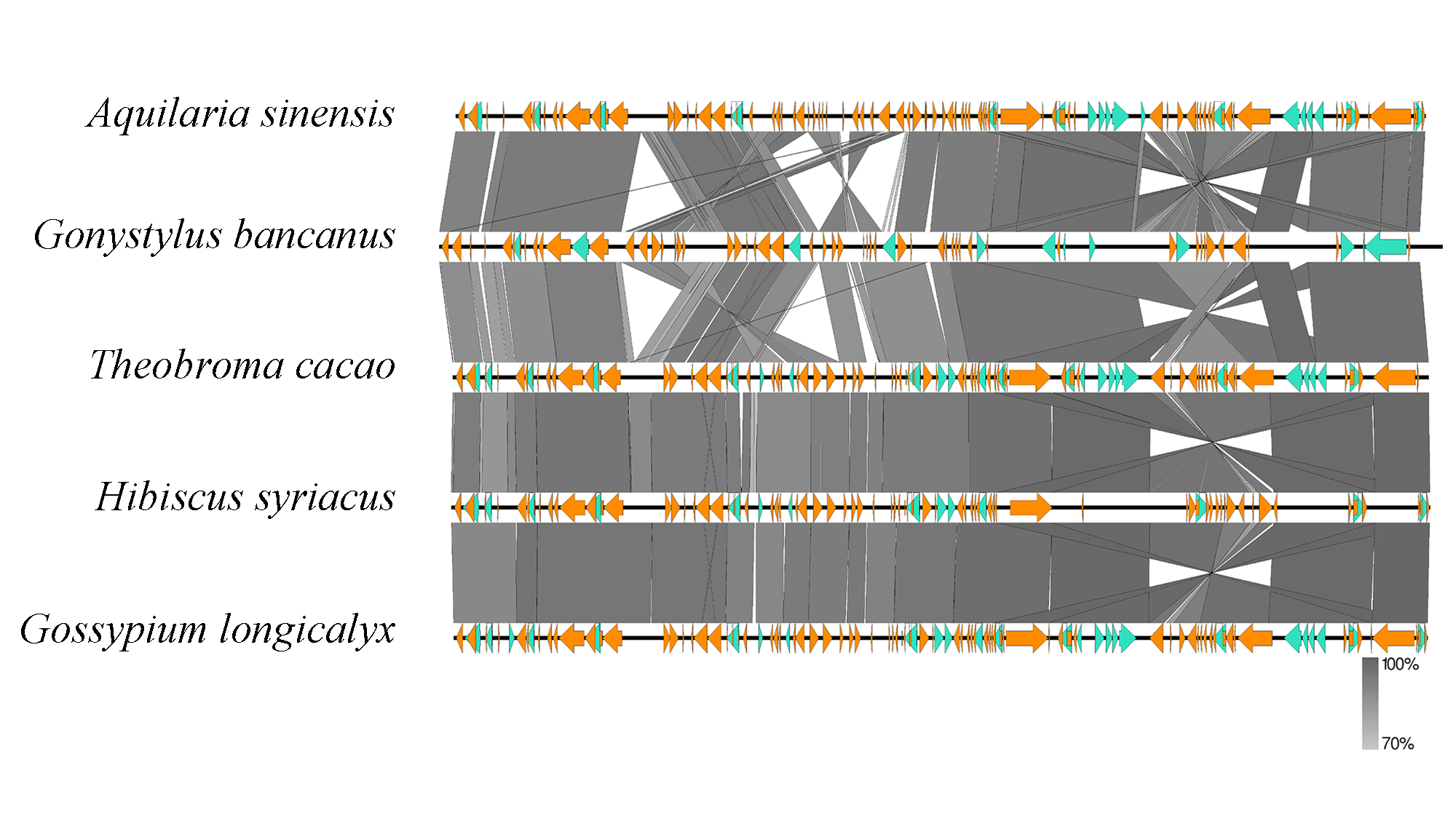

Supplement: Figure S2 — Comparisons of CP genomic structure among five land plants, including A. sinensis, using Easyfig. The arrowheads indicate genes with their orientations and positions. The orange arrows indicate the coding regions of genes. Gray ligature indicate positions and similarity of the genes of five land plants. [file Image2.TIF]
