# Supplementary material for: Complete Chloroplast Genome Sequence of Aquilaria sinensis (Lour.) Gilg and Evolution Analysis within the Malvales Order
Source: Front Plant Sci. 2016 Mar 8;7:280. doi: 10.3389/fpls.2016.00280 (PMC4781844; doi:10.3389/fpls.2016.00280)
Supplement: Table S1 — List of CP genomes used as reference to map to contigs. [file Table1.DOCX]

| **Accession** | **species** | **genome.size** | **GC.** | **#gene** |
| --- | --- | --- | --- | --- |
| EU849490.1* | Gonystylus bancanus | 165110 | 37.3048 | 72 |
| NC_026909.1* | Hibiscus syriacus | 161019 | 36.8298 | 100 |
| NC_011163.1 | Cicer arietinum | 125319 | 33.9063 | 108 |
| NC_023959.1 | Trigonobalanus doichangensis | 159938 | 36.9893 | 120 |
| NC_024747.1 | Populus euphratica | 156766 | 36.7146 | 123 |
| NC_014674.1 | Castanea mollissima | 160799 | 36.7639 | 124 |
| NC_008325.1 | Daucus carota | 155911 | 37.6555 | 126 |
| NC_008334.1 | Citrus sinensis | 160129 | 38.4821 | 126 |
| NC_014676.2 | Theobroma cacao | 160619 | 36.8742 | 128 |
| NC_024734.1 | Populus fremontii | 157446 | 36.6748 | 129 |
| NC_024735.1 | Populus balsamifera | 157094 | 36.6507 | 129 |
| NC_015206.1 | Fragaria vesca | 155691 | 37.2128 | 130 |
| NC_022392.1 | Eucalyptus melliodora | 160386 | 36.8436 | 130 |
| NC_022396.1 | Eucalyptus aromaphloia | 160149 | 36.8607 | 130 |
| NC_022401.1 | Eucalyptus torquata | 160223 | 36.8636 | 130 |
| NC_022414.1 | Stockwellia quadrifida | 159561 | 36.8511 | 130 |
| NC_024060.1 | Hirtella racemosa | 162891 | 36.2347 | 130 |
| NC_024061.1 | Chrysobalanus icaco | 162775 | 36.1732 | 130 |
| NC_024062.1 | Licania heteromorpha | 162833 | 36.2015 | 130 |
| NC_024066.1 | Hirtella physophora | 162955 | 36.2474 | 130 |
| NC_007942.1 | Glycine max | 152218 | 35.373 | 131 |
| NC_010433.1 | Manihot esculenta | 161453 | 35.873 | 131 |
| NC_012224.1 | Jatropha curcas | 163856 | 35.3579 | 131 |
| NC_016736.1 | Ricinus communis | 163161 | 35.742 | 131 |
| NC_023214.1 | Gossypium bickii | 159422 | 37.1969 | 134 |
| NC_015308.1 | Hevea brasiliensis | 161191 | 35.7439 | 136 |
| NC_022813.1 | Schefflera delavayi | 156341 | 37.8295 | 136 |
| NC_023216.1 | Gossypium longicalyx | 160241 | 37.242 | 136 |
| NC_021425.1 | Tetracentron sinense | 164467 | 38.1402 | 140 |
| NC_024929.1 | Citrus aurantiifolia | 159893 | 38.4395 | 140 |
| *: incomplete annotation | | | | |

**Table S1: The list of CP genomes that were used as reference to map to contigs.**
