# Supplementary material for: Complete Chloroplast Genome Sequence of Aquilaria sinensis (Lour.) Gilg and Evolution Analysis within the Malvales Order
Source: Front Plant Sci. 2016 Mar 8;7:280. doi: 10.3389/fpls.2016.00280 (PMC4781844; doi:10.3389/fpls.2016.00280)
Supplement: Table S2 — Summary of A. sinensis chloroplast genome characteristics. [file Table2.DOCX]

**Table S2. Summary of *A. sinensis* chloroplast genome characteristics.**

| **Attribute** |  | **A(U)(%)** | **T(%)** | **G(%)** | **C(%)** | **GC（%）** | **Length(bp)** |
| --- | --- | --- | --- | --- | --- | --- | --- |
| LSC |  | 32.04 | 33.01 | 16.73 | 18.22 | 34.95 | 87482 |
| IRA |  | 28.60 | 28.54 | 22.29 | 20.57 | 42.86 | 26113 |
| SSC |  | 34.52 | 33.91 | 15.09 | 16.49 | 31.58 | 19857 |
| IRB |  | 28.54 | 28.60 | 20.58 | 22.28 | 42.86 | 26113 |
| Total |  | 31.21 | 31.68 | 18.06 | 19.05 | 37.11 | 159565 |
| CDS |  | 30.58 | 31.52 | 20.21 | 17.70 | 37.90 | 79830 |
|  | 1st position | 30.50 | 24.13 | 26.45 | 18.91 | 45.36 | 26610 |
|  | 2st position | 29.78 | 32.53 | 17.68 | 20.01 | 37.69 | 26610 |
|  | 3st position | 31.45 | 37.89 | 16.49 | 14.17 | 30.66 | 26610 |
