# Supplementary material for: Complete Chloroplast Genome Sequence of Aquilaria sinensis (Lour.) Gilg and Evolution Analysis within the Malvales Order
Source: Front Plant Sci. 2016 Mar 8;7:280. doi: 10.3389/fpls.2016.00280 (PMC4781844; doi:10.3389/fpls.2016.00280)
Supplement: Table S3 — The genes in the A. sinensis chloroplast genome with introns. [file Table3.DOCX]

**Table S3 The genes in the *A. sinensis* chloroplast genome with introns.**

| Gene | Location | Exon I (bp) | Intron I (bp) | Exon II (bp) | Intron II (bp) | Exon III (bp) |
| --- | --- | --- | --- | --- | --- | --- |
| rpoC1 | LSC | 1637 | 750 | 466 |  |  |
| ycf3 | LSC | 153 | 742 | 230 | 734 | 127 |
| atpF | LSC | 476 | 791 | 163 |  |  |
| ndhA | SSC | 539 | 1148 | 574 |  |  |
| tRNA-Ala | IR | 38 | 818 | 35 |  |  |
| tRNA-Ile | IR | 35 | 956 | 42 |  |  |
| ndhB | IR | 756 | 589 | 870 |  |  |
| rpl2 | IR | 470 | 648 | 394 |  |  |
| ndhB* | IR | 777 | 682 | 756 |  |  |
| rpl2* | IR | 391 | 651 | 470 |  |  |
