# Supplementary material for: Complete Chloroplast Genome Sequence of Aquilaria sinensis (Lour.) Gilg and Evolution Analysis within the Malvales Order
Source: Front Plant Sci. 2016 Mar 8;7:280. doi: 10.3389/fpls.2016.00280 (PMC4781844; doi:10.3389/fpls.2016.00280)
Supplement: Table S4 — Relative synonymous codon usage (RSCU) for the A. sinensis chloroplast genome. [file Table4.DOCX]

**Table S4 Relative synonymous codon usage(RSCU) for the *A. sinensis* chloroplast genome.**

| Codon | AA | Number | Fraction | Frequency | RSCU |
| --- | --- | --- | --- | --- | --- |
|  |  |  |  |  |  |
|  |  |  |  |  |  |
| AAA | K | 1056 | 0.714 | 40.367 | 1.2785882 |
| AAC | N | 284 | 0.228 | 10.856 | 0.4955294 |
| AAG | K | 424 | 0.286 | 16.208 | 0.4861176 |
| AAT | N | 963 | 0.772 | 36.812 | 1.3162353 |
| ACA | T | 384 | 0.306 | 14.679 | 1.0925882 |
| ACC | T | 225 | 0.179 | 8.601 | 0.7236471 |
| ACG | T | 129 | 0.103 | 4.931 | 0.5327059 |
| ACT | T | 516 | 0.411 | 19.725 | 1.6035294 |
| AGA | R | 492 | 0.317 | 18.807 | 1.8129412 |
| AGC | S | 137 | 0.068 | 5.237 | 0.4376471 |
| AGG | R | 174 | 0.112 | 6.651 | 0.5345882 |
| AGT | S | 392 | 0.194 | 14.985 | 1.266 |
| ATA | I | 670 | 0.304 | 25.612 | 0.8851765 |
| ATC | I | 439 | 0.199 | 16.781 | 0.6216471 |
| ATG | M | 591 | 1 | 22.592 | 0.9411765 |
| ATT | I | 1097 | 0.497 | 41.934 | 1.4929412 |
| CAA | Q | 714 | 0.759 | 27.294 | 1.3697647 |
| CAC | H | 148 | 0.236 | 5.657 | 0.3877647 |
| CAG | Q | 227 | 0.241 | 8.677 | 0.4184706 |
| CAT | H | 480 | 0.764 | 18.349 | 1.1651765 |
| CCA | P | 296 | 0.272 | 11.315 | 1.0123529 |
| CCC | P | 217 | 0.199 | 8.295 | 0.7304706 |
| CCG | P | 148 | 0.136 | 5.657 | 0.5305882 |
| CCT | P | 429 | 0.394 | 16.399 | 1.4904706 |
| CGA | R | 339 | 0.219 | 12.959 | 1.4291765 |
| CGC | R | 116 | 0.075 | 4.434 | 0.3785882 |
| CGG | R | 116 | 0.075 | 4.434 | 0.3623529 |
| CGT | R | 314 | 0.202 | 12.003 | 1.3417647 |
| CTA | L | 388 | 0.137 | 14.832 | 0.7456471 |
| CTC | L | 200 | 0.071 | 7.645 | 0.5297647 |
| CTG | L | 182 | 0.064 | 6.957 | 0.3736471 |
| CTT | L | 609 | 0.216 | 23.28 | 1.1812941 |
| GAA | E | 993 | 0.731 | 37.959 | 1.3188235 |
| GAC | D | 218 | 0.201 | 8.333 | 0.3257647 |
| GAG | E | 366 | 0.269 | 13.991 | 0.3988235 |
| GAT | D | 864 | 0.799 | 33.028 | 1.5095294 |
| GCA | A | 384 | 0.277 | 14.679 | 1.0892941 |
| GCC | A | 225 | 0.162 | 8.601 | 0.6832941 |
| GCG | A | 161 | 0.116 | 6.154 | 0.4284706 |
| GCT | A | 617 | 0.445 | 23.586 | 1.7042353 |
| GGA | G | 682 | 0.394 | 26.07 | 1.5734118 |
| GGC | G | 165 | 0.095 | 6.307 | 0.4008235 |
| GGG | G | 321 | 0.185 | 12.271 | 0.7111765 |
| GGT | G | 565 | 0.326 | 21.598 | 1.2197647 |
| GTA | V | 506 | 0.372 | 19.343 | 1.5267059 |
| GTC | V | 165 | 0.121 | 6.307 | 0.4817647 |
| GTG | V | 191 | 0.14 | 7.301 | 0.5161176 |
| GTT | V | 499 | 0.367 | 19.075 | 1.4752941 |
| TAA | * | 37 | 0.435 | 1.414 | 1.3058824 |
| TAC | Y | 190 | 0.192 | 7.263 | 0.3232941 |
| TAG | * | 26 | 0.306 | 0.994 | 0.9176471 |
| TAT | Y | 798 | 0.808 | 30.505 | 1.5825882 |
| TCA | S | 386 | 0.191 | 14.755 | 1.0734118 |
| TCC | S | 313 | 0.155 | 11.965 | 0.9235294 |
| TCG | S | 192 | 0.095 | 7.339 | 0.5647059 |
| TCT | S | 604 | 0.298 | 23.089 | 1.6648235 |
| TGA | * | 22 | 0.259 | 0.841 | 0.7764706 |
| TGC | C | 86 | 0.279 | 3.287 | 0.3223529 |
| TGG | W | 473 | 1 | 18.081 | 0.7764706 |
| TGT | C | 222 | 0.721 | 8.486 | 1.1364706 |
| TTA | L | 856 | 0.303 | 32.722 | 1.9117647 |
| TTC | F | 575 | 0.371 | 21.98 | 0.6861176 |
| TTG | L | 587 | 0.208 | 22.439 | 1.2587059 |
| TTT | F | 975 | 0.629 | 37.271 | 1.2903529 |
|  | | | | | |
|  | | | | | |
